# Quantification of dolichol in the human lens with different types of cataracts

**Published:** 2009-08-11

**Authors:** Devarshi Gajjar, Adam Jozwiak, Ewa Swiezewska, Bhagwat Alapure, Trilok Parmar, Kaid Johar, Abhay R. Vasavada

**Affiliations:** 1 Iladevi Cataract and IOL Research Centre, Ahmedabad, India; 2 Department of Lipid Biochemistry, Polish Academy of Sciences, Warsaw, Poland

## Abstract

**Purpose:**

To quantify and characterize dolichol species in cataractous and clear human lenses.

**Methods:**

Whole lenses were collected from cadaver eyeballs from the C.H. Nagri Eye Bank and Red Cross Society Eye Bank (Ahmedabad, India). Cataractous nuclei were collected after extracapsular cataract extraction (ECCE). Wet weight for all the lenses was taken and were stored at –50 °C until used. Dolichol was extracted using a standard protocol and then analyzed using High Performance Liquid Chromatography (HPLC) on a 4.6 mm×60 mm Hypersil-Octadecylsilane (ODS; 3 μm) reversed phase column using a Waters dual pump apparatus, a Waters gradient programmer, and an ultraviolet (UV) detector set at 210 nm. Dolichol 13 was used as an internal standard, and dolichol mixture from the liver was used as an external qualitative standard.

**Results:**

The highest dolichol concentration was found in nuclear cataract (2.54±0.6 μg) followed by posterior subcapsular cataract (1.4±0.35 μg), and the lowest levels were observed in cortical cataract (0.37±0.06 μg). The level of dolichol concentration in cataractous lenses was statistically significantly higher than the levels in clear lenses (1.0±04.3 μg; p<0.01).

**Conclusions:**

The dolichol concentration was significantly higher in lenses with nuclear cataract. A significant difference in dolichol concentration was observed between the different types of cataract. It suggests that dolichol and other isoprenoids may be associated with cataractogenesis.

## Introduction

Dolichols are long chains of hydrophobic lipids broadly distributed in all tissues and cellular membranes of eukaryotic cells [1]. The biological role of phosphorylated dolichols as cofactors in the biosynthesis of glycoproteins is well characterized [2]. Dolichols are also postulated to serve as donors of isoprenoid groups during protein prenylation. Biophysical studies have shown that dolichols exert an effect on the permeability and fluidity of model membranes [3]. In line with this observation was the finding that dolichol is involved in the transport of the endoplasmic reticulum (ER) and vacuolar proteins in yeast [4]. With reference to the locations and stoichiometry of polyunsaturated fatty acids (PUFA), dolichol and vitamin E, it is proposed that these molecules interact with each other to form a highly matched free radical transfer chain whose malfunctioning could be involved in statin toxicity and neurodegenerative diseases [5]. Hence, dolichol most probably affects the structure and fluidity of the cellular membrane and consequently has an influence on the activity of membrane-associated proteins. It also seems plausible to presume that dolichol protects the components of biological membranes against oxidative stress [5]. Dolichol has also been shown to delay the G_1_ cell cycle arrest in human fibroblasts [6]. On the other hand, plant isoprenoids have been shown to initiate apoptosis and cell cycle arrest in murine tumor cells, which in turn suppress the growth of the tumor [7]. The isoprenoid-mediated induction of detoxifying activities and the antioxidant activity of some plant isoprenoids is well recognized [8]. Thus, dolichol seems critical for many important events in cellular life.

The lens, an encapsulated structure, consists of a large number of elongated cells also known as fiber cells. They are produced via terminal differentiation of a monolayer of epithelial cells lining the anterior lens capsule. During terminal differentiation, fiber cells lose all subcellular organelles and so the plasma membrane becomes essentially the only organelle of the lens. This phenomenon makes the lens one of the best tissues to study the fundamental aspects of cell differentiation and aging. Our interest in dolichol originated from the possibility that there could be a connection between dolichol and cataract, as cataract is an age-related disease and dolichol is a marker for aging [1]. Moreover, oxidative stress is the major risk factor for cataract, and dolichol has been proposed to protect membrane lipids against oxidative free radical species and it also has ultraviolet (UV) absorbing properties [9]. Since it has been observed that dolichol is altered under conditions of oxidative stress [10], we decided to quantitate dolichol in human lenses with and without cataract.

The presence of dolichol in the lens is constitutive, and hence it may be required in the normal functioning of the lens fiber cell membrane. In the present study, dolichol levels in human lenses from different types of cataract were measured. Further, we also tried to quantitate different dolichol species. To our knowledge, the present study is the first of its kind to characterize dolichol species in the human lens and to define its structural role in the lens in association with its pathological condition.

## Methods

### Collection of human lens samples

Whole lenses (clear) were collected from cadaver eyeballs from the C. H. Nagri Eye Bank and Red Cross Society Eye Bank (Ahmedabad, India). Cataractous nuclei were collected after extracapsular cataract extraction (ECCE). All the lenses were stored at –50 °C until use. All the lenses collected from human donor eyes (postmortem time 4–12 h) were in accordance with the Declaration of Helsinki. Ethical approval for the use of human lenses was obtained from the Institutional Ethics Committee of ICIRC.

Cataract patients between 45 and 70 years with pupils dilating more than 7 mm and with otherwise normal eyes were included in the study. Exclusion criteria were diabetes mellitus, hypertension, glaucoma, shallow anterior chamber, uveitis, high myopia (axial length >27.0 mm), pseudoexfoliation, traumatic cataract, subluxated cataract, previous ocular surgeries, ocular disease, patients on steroids or immunosuppressive therapy, and those allergic to dilating drops. All the patients were subjected to a slit-lamp examination, and the type of cataract was recorded according to the zone of opacification (LOCs III Classification System [11]). The grade of cataract was also recorded based on the degree of hardness using the Emery and Little classification [12].

### Quantification of dolichol

#### Extraction of dolichol from the lens

Wet weight of the lenses was taken, and lenses were stored at −50 °C until further use. Dolichol extraction was done using the method of Fedorow et al. [13]. Briefly, the lens tissue supplemented with an internal standard (0.25 μg of dolichol 13 [Dol-13]) was homogenized in one volume of methanol and extracted with five volumes of hexane. The hexane phase was separated after low speed centrifugation and then dried under nitrogen gas. The dried lipids were re-dissolved in a known volume of isopropanol and stored at −50 °C for later HPLC analysis.

#### HPLC-UV quantification of total dolichol from the lens lipid extract

All HPLC analysis was performed as described by Skorupinska-Tudek et al. [14].

Analysis of extracted polyisoprenoids was performed using a Waters dual pump apparatus (Waters Corporation, Milford MA),  a Hypersil ODS column (4.6 mm×60 mm, 3 μm; Knauer, Berlin, Germany), a Waters gradient programmer, and a UV detector set at 210 nm. For elution, a combination of convex (Waters no. 5, from 0 to 75% B for the initial 20 min; where solvent A was methanol/water, 9:1, vol/vol, and solvent B was methanol/propan-2-ol/hexane, 2:1:1, by volume) and linear (from 75 to 100% B during the following 10 min) gradients was used; in the last 5 min, re-equilibration back to 0% B was performed. The solvent flow rate was 1.5 ml/min. The amount of dolichols was estimated by comparison with an internal standard of Dol-13. The chain length and identity of lipids was confirmed by applying qualitative standards extracted from the human liver containing a mixture of dolichols (Dol-17, -18, -19, -20, and -21).

Statistical analysis was performed using non-parametric tests namely the Kruskal–Wallis test and the Mann–Whitney U tests. A value of p < 0.05 was considered significant.

### Chemicals and standards

Organic solvents (HPLC grade) were purchased from Merck, Ltd. (Mumbai, India) and all the other chemicals (p.a. grade) were from Sigma-Aldrich (St. Louis, MO). Standards of dolichol were from the ‘Collection of Polyprenols’ at the Department of Lipid Biochemistry (Institute of Biochemistry and Biophysics Polish Academy of Sciences, Warsaw, Poland).

## Results

Table 1 shows the mean and median age of the patients based on the type of cataract. There was no statistically significant difference in the age of patients with different types of cataract. Table 2 shows the dolichol content in the human lenses of different types of cataract. The level of dolichol in clear lenses was 1.0±0.43 μg/g (wet weight). The level of dolichol in lenses with nuclear cataract was 2.54±0.60 μg/g (wet weight). In those with cortical cataract, it was 0.37±0.06 μg/g (wet weight). Thus, compared to clear lenses, the dolichol level in eyes with nuclear cataract was 2.5 times more while in eyes with cortical cataract it was 2.7 times less. The difference was statistically significant (p=0.004 for nuclear and p=0.01 for cortical cataract).

**Table 1:** Age of the patients in each type of cataract.

**Cataract type**	**Mean age (years) ±SD**
Non-cataractous (n=7)	51±10.1
Nuclear (Type 1; n=6)	62.66±4.36
Cortical (Type 2,3; n=6)	46.3±7.6
Posterior Subcapsular (Type 4; n=6)	57.3±8.6
White mature cataract (n=6)	64.3±7.11

The age of cataract patients included in the study ranged between 45 and 70 years. Values are presented as  mean±SD . There was no statistically significant difference in the age of patients with different types of cataract.

**Table 2:** Dolichol concentration in the human lens.

**Cataract type**	**Dolichol content (μg/g fresh weight)**		**p value**
	**mean value**	**median value**	
Control (Non-cataractous)	1.00±0.43	0.99	
Nuclear	2.54±0.60	2.53	0.004*
Cortical	0.37±0.060	0.37	0.015*
Posterior Subcapsular	1.42±0.35	1.39	0.13
White mature cataract	1.47±0.36	1.45	0.06

The asterisk indicates that there was a statistically significant difference. There was a marked variability in the concentration of total dolichol in lens of different types of cataract. The highest levels of dolichol concentration were found in nuclear cataract followed by posterior subcapsular cataract, and the lowest levels were observed in cortical cataract. The dolichol concentration in cataractous lenses was statistically significantly higher than the levels in non-cataractous lenses (p<0.01).

**Figure 1:**
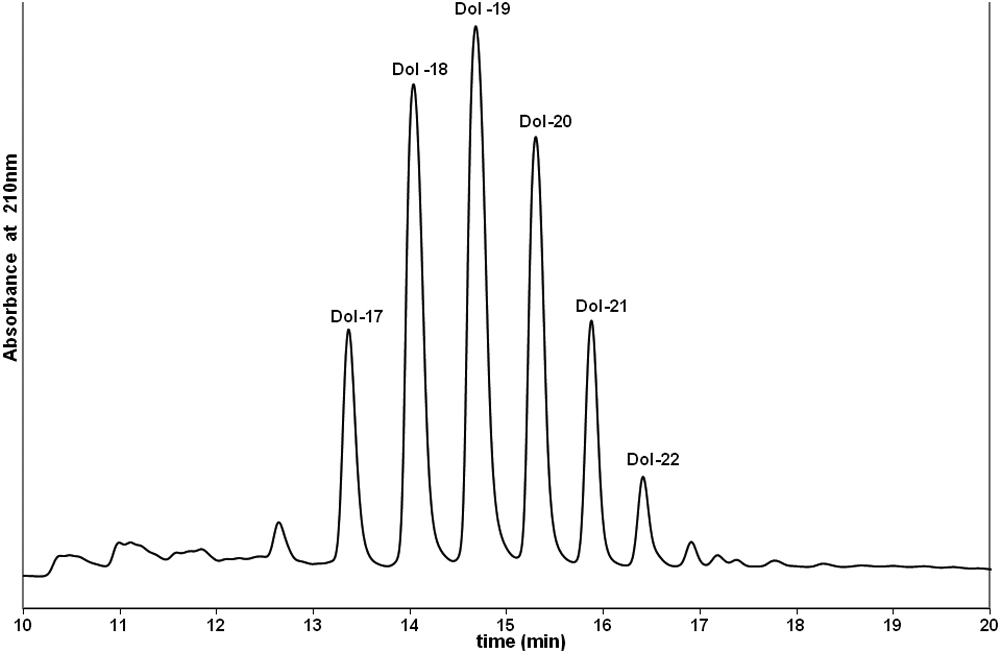
HPLC profile standard dolichol extracted from the human liver. Profile was obtained by monitering the elute at 210 nm. The molecular species distribution of standard dolichols show dolichols ranging from Dol-17 to Dol-22.  The numbers in the chromatogram identify the six major well defined peaks that are of individual dolichol species corresponding to Dol-17, Dol-18, Dol-19, Dol-20, Dol-21, and Dol-22.

**Figure 2:**
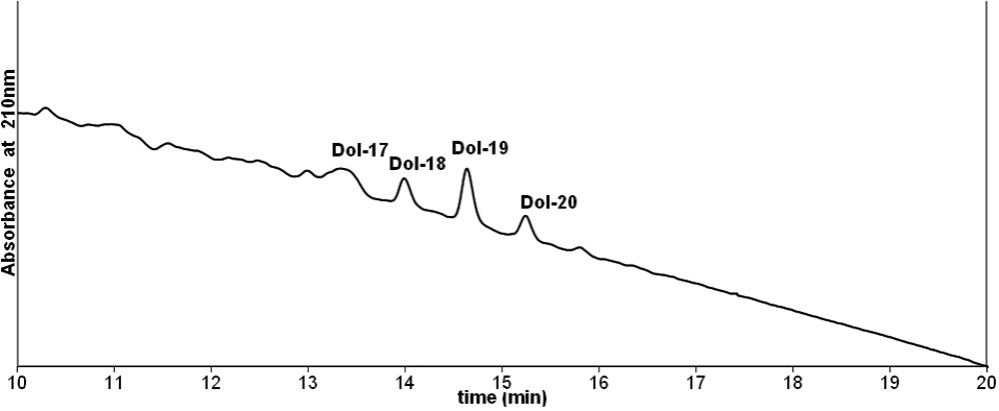
HPLC profile of total dolichol extracted from clear human lens. The peaks show the molecular species distribution of dolichols in normal lenses where dolichols ranging from 17 to 20 isoprene units were detected. The most abundant species is the Dol-19 component.

**Figure 3:**
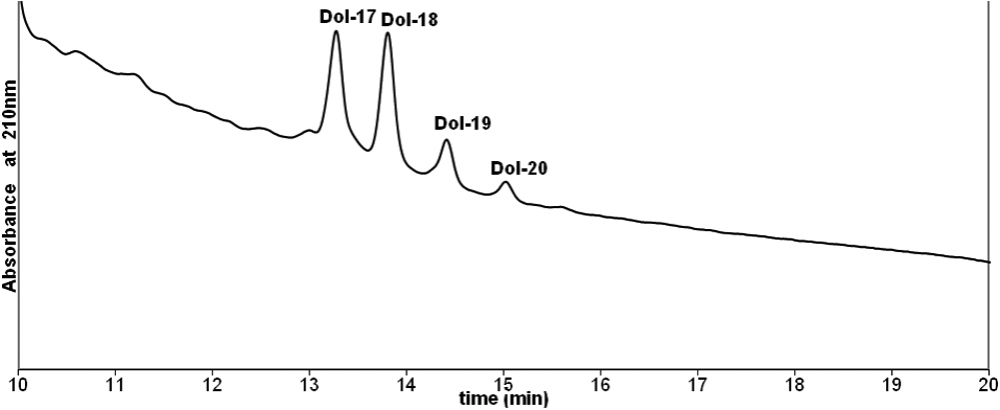
HPLC profile of total dolichol extracted from human lens with nuclear cataract. The peaks show the molecular species distribution of dolichols. A shift in the dolichol chain length toward the shorter chain length was observed in nuclear cataractous lenses. The range of species was the same as those for the clear lenses. The most abundant species was Dol-18 instead of Dol-19.

**Figure 4:**
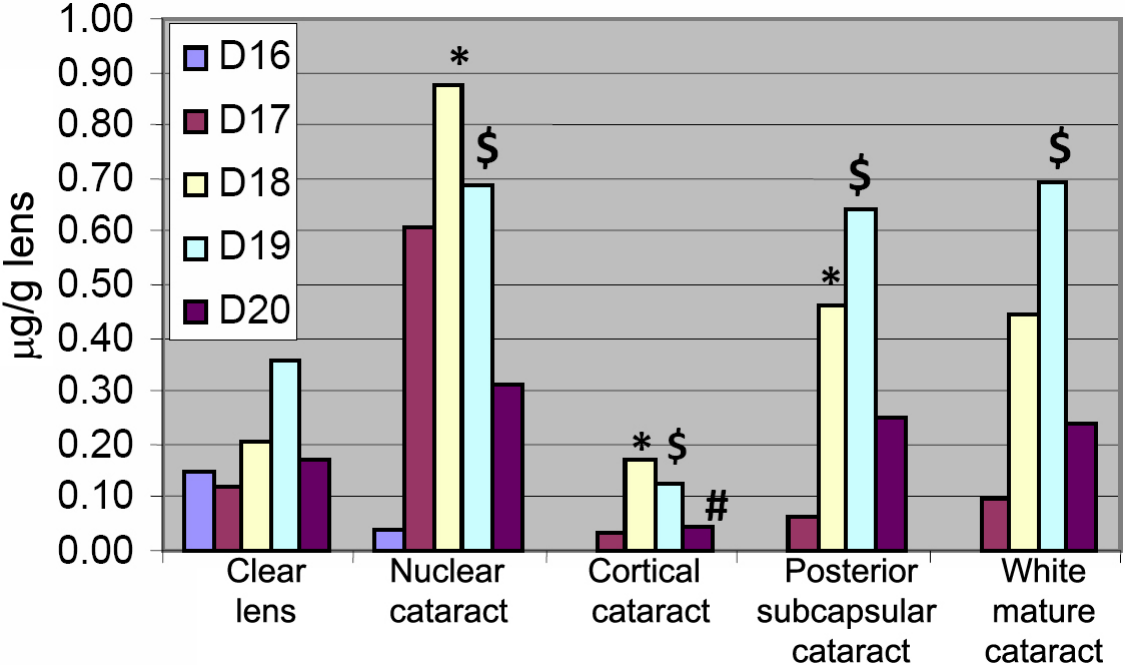
Concentration of each dolichol species in lenses with different types of cataract. A statistically significant increase in both Dol-18 and Dol-19 concentrations compared to clear lenses was found in nuclear cataract and posterior subcapsular cataract. In contrast, a statistically significant decrease in Dol-19 and Dol-20 content was found in cortical cataract. An asterisk indicates that p=0.002 when compared to clear lens. The dollar sign ($) denotes that p=0.015 when compared to clear lens, and a hash mark means p=0.03 when compared to clear lens.

Figure 1 shows the molecular species distribution of standard dolichols extracted from the human liver. Figure 2 shows the molecular species distribution of dolichols in clear lenses where dolichols ranging from 16 to 20 isoprene units were detected. The most abundant species is the 19 isoprene component. Figure 3 shows the molecular species distribution of dolichols obtained from nuclear cataractous lenses. A slight shift in the dolichol chain length toward the shorter chain length was observed in nuclear and cortical cataractous lenses. The range of species was the same as those for the clear lenses. However, the most abundant species was Dol-18 instead of Dol-19. In nuclear cataract, there was a statistically significant increase in both Dol-18 and Dol-19 concentration compared to clear lenses (p=0.002 and p=0.01). Figure 4 shows the comparison of each dolichol species in different types of cataract using the Mann–Whitney U test. The intact normal clear adult human lenses contained between 0.80–1.00 μg/g (wet weight) of dolichol. Out of this, 15% was Dol-16, 12% was Dol-17, 21% was Dol-18, 36% was Dol-19, and 17% was Dol-20. Further, lenses with nuclear cataract contained 2.54 μg/g of dolichol of which 1.5% was Dol-16, 24% was Dol-17, 34% was Dol-18, 27% was Dol-19, and 12.2% was Dol-20. Contrary to nuclear cataract, the statistically significant increase in dolichol content in posterior subcapsular cataract was due to the significant increase in Dol-18 and Dol-19 concentration (p=0.03 and p=0.004, respectively). However, in white mature cataract, there was a significant increase only in Dol-19 concentration (p=0.004) and not in any other dolichol species. In cortical cataract, there was a significant decrease in Dol-19 and Dol-20 content (p=0.009 and p=0.02, respectively). Similarly, the total dolichol content decreased in cortical cataract, and the distribution was 10.8% of Dol-17, 45.9% of Dol-18, 35% of Dol-19, and 10.8% of Dol-20.

## Discussion

In the present study, whole clear lenses from cadaver eyes used for analysis were considered control samples, and the nucleus obtained after ECCE was used for lenses from different types of cataracts. The reason for this is because of the limitation of the extraction technique. In the clear lens, the nucleus is not well defined and so cannot be extracted in the same way as the cataractous nucleus.

The highest dolichol concentration was found in lenses with nuclear cataract followed by white mature cataract and posterior subcapsular cataract whereas dolichol concentration was lower in lenses with cortical cataract when compared to clear lenses. Nuclear cataract or nuclear sclerosis is considered to be an age-related cataract, and oxidative stress is a well established risk factor for age-related cataract. It is suggested that nuclear cataract is the endpoint of several events generated due to the effect of oxidative stress, which includes a decrease in antioxidative enzymes, lipid peroxidation, and protein precipitation. The effects of oxidative stress on the dolichol content were recently studied on isolated rat liver cells by Guarini et al. [10]. Oxidative stress caused an increase in lipid peroxidation and rapid degradation of dolichol probably by the same (unknown) mechanism responsible for the breakdown of ubiquinone isoprenoid chains. The oxidized derivative of dolichol, dolichoic acid, was reported in mammalian tissues, i.e., in bovine thyroid [15] and neuromelanin. In parallel, enzymes responsible for the conversion of dolichol to dolichal and dolichoic acid were also described [16,17]. In the present study, the concentration of Dol-18 increased over that of Dol-19 in cataractous lenses. However, further work is required to analyze if dolichoic acid is formed in cataractous lenses.

Dolichol concentration in lenses with cortical cataract was 2.7 fold lower than in clear lenses. Despite the lack of knowledge on the underlying mechanism leading to the development cortical cataract, epidemiological studies suggest that prolonged exposure to sunlight [18], UV irradiation [19], diabetes [20], and vascular diseases [20] play a vital role in this process. Treatment of UV radiation caused a dramatic depletion of dolichol levels in a culture model using liver parenchymal cells [21]. After in vitro UV radiation, fragmentation of dolichol was observed [21]. Hence, it is not surprising that the dolichol content is less in cortical cataract. Whether such a non-enzymatic mechanism is responsible for the degradation of dolichol in UV-exposed human lenses remains to be established. While oxidative stress continues to play an important role in nuclear cataract in general, its role in the formation of cortical cataract has not been substantiated. Cortical cataract is shown to be due to a local loss of osmotic regulation and calcium overload [22]. Hence, there is adequate evidence showing the differences in etiology for the two types of cataracts [22,23]. In contrast, there was an increase in dolichol content in posterior subcapsular cataract and white mature cataract, although no shift in the chain length was seen. In fact, the increase in dolichol levels was mainly due to the increase in dolichol 19 levels.

What are the consequences of the increased or decreased concentration of dolichols in human lenses? Since this is the first report on dolichol concentration, it is difficult to arrive at any general conclusion. The accumulation of dolichols in membranes could destabilize them as these compounds are fusogenic and can alter fluidity, membrane permeability, and membrane functioning [2]. Oxidation of lenticular membranes and cytosolic components is believed to lead to cataractogenesis in humans [23]. Hence, there has been great interest in the natural antioxidants of lenses and the possibility of delaying cataract development [24,25]. So far the only human endogenous lipid antioxidant reported in the lens is ubiquinone (coenzyme Q) with a concentration of 0.3–0.4 μg/g [26]. The ubiquinone molecule consists of a polyisoprene hydrocarbon chain built of 10 isoprene units attached to a parabenzoquinone ring. Dolichol levels measured in the lenses in the present study were three times higher than ubiquinone levels. However, it is unclear whether increased dolichol levels are observed in nuclear cataract because of the altered cholesterol synthetic pathway as seen in cataract formation or if dolichol acts as a free radical scavenger as postulated earlier [5]. However, the potential mechanism of dolichol involvement in cataract etiology needs to be addressed.

The change in the distribution of free dolichol in human cataract is of interest. In clear lenses, Dol-18, -19, and -20 dominate with Dol-19 exceeding Dol-18 and Dol-20 1.7 and 2.2 fold, respectively. However, in nuclear cataractous lenses, Dol-18 exceeds Dol-19 and Dol-20 1.2 and 2.8 fold, respectively. In all the human tissues studied so far, Dol-19 was the predominant species [27]. Thus the lipid distribution in cataractous lenses seems to have shifted in favor of slightly lower lengths and a more narrow distribution (Figure 2). The dolichol chain length pattern is considered a species specific marker. It does not vary in different tissues of animals belonging to the same species [28,29]. The effect of pathological conditions on the dolichol chain length is not understood. Eggens et al. [30] noted that the chain length of the dominating dolichol isolated from an autopsy of human hepatocarcinoma samples was shorter by one isoprene unit as compared to that observed in normal liver cells, i.e., there was a relative increase in the content of Dol-18 and 17 together with a decrease in the content of Dol-20 and Dol-21. These observations were explained by the preferential use of farnesyl diphosphate, a common precursor of cholesterol, dolichol, and ubiquinone, for the biosynthesis of cholesterol. In contrast, Sagami et al. [31] proposed that the formation of the shorter chain length product was due to the limited concentration of the polymerizing monomeric substrate isopentenyl diphosphate. In contrast, a shift toward longer dolichol species has been reported in preneoplastic nodules [32] in rat liver cells. The mechanism responsible for the modulation of the product specificity of cis-prenyltransferase, the enzyme catalyzing the synthesis of the dolichol chain, remains unclear. In the present study, dolichols in all forms are higher in cataractous lenses than in the control. Although we are unable to explain this, we speculate three possibilities: (1) an increase in the amount of total dolichol by the preferential use of farnesyl diphosphate, a common precursor of cholesterol, dolichol and ubiquinone; (2) possible conversion of dol-19 to the smaller species; and (3) modulation of the activity of cis-prenyltransferase.

Dolichol may function in the lens as a free radical protecting agent and/or a modulator of physicochemical properties of the membrane [2,33]. The tissue concentration of dolichol and its derivatives has been investigated in several pathological conditions, and considerable changes have been described. The levels of dolichol increased during aging, cirrhosis, liver cancer, and ceroid lipofuscinosis, e.g., dolichol levels increased in human and rat tissues upon aging [34]. A sevenfold increase in dolichol levels was observed in the lungs, and a 150 fold increase was observed in the pancreas with age. An age-dependent increase in dolichol concentration has been documented in many tissues such as the liver, heart, brain, kidney, etc. [2,33]. On the other hand, no change has been documented in dolichol levels in tissues like the spleen. Contrary reports have been published on an increase as well as a decrease in plasma dolichol concentrations [35]. In the present study, no obvious correlation was observed between dolichol levels and the age of the lenses. However, the sample size was too small to draw a general conclusion.

To our knowledge, there have been no reports on the presence of dolichol in human lenses, although prenylation of lens proteins has been reported [36].

The accumulation of dolichol in the lens might possibly influence the process of glycosylation of proteins since dolichyl phosphate acts as a cofactor in this process. Although human tissues possess the appropriate enzymatic machinery, dolichyl kinase [37], its expression in the lens has not yet been analyzed.

In general, more than 70% of all the proteins in eukaryotic cells are predicted to be N-glycosylated [38]. In this regard, regional differences have been reported in glycoprotein expression in lens epithelial cells and fibers of chicken lenses [39]. This indicates that glycoproteins play a role in the regulation of lens functioning.

The composition of lipids in lenses has been extensively studied [40]. Lens membranes have the highest cholesterol content of any known membrane, but there appears to be no clear relationship between lens cholesterol content and human senile cataract [41]. Inherited defects in enzymes involved in cholesterol metabolism and the use of drugs, which inhibit lens cholesterol biosynthesis, can be associated with cataract formation in animals and humans [41]. Out of the six inborn defects of cholesterol biosynthesis described in humans, three defects namely mevalonic aciduria [42], Smith-Lemli-Opitz syndrome [43], and X-linked dominant chondroplasia punctata 2 [44] have cataract as complications. The cause of cataract in these diseases was postulated to be due to the decrease in cholesterol levels in lenses. Dolichol, however, has not been analyzed in such conditions.

Based on all these facts and hypotheses, it would be interesting to analyze the differences in glycosylated proteins of lenses. Furthermore, it would be interesting to estimate the levels of key enzymes of the mevalonate pathway, e.g., 3-hydroxy-3-methyl-glutaryl-CoA (HMG CoA) reductase, cis*-*prenyl transferase, etc.

In conclusion, a significant difference in dolichol concentration was observed between the different types of cataract with the highest concentration in lenses with nuclear cataract and the lowest in cortical cataract, suggesting that dolichol and other isoprenoids may be associated with cataractogenesis.
